# Antimicrobial efficacy of commercially available endodontic bioceramic root canal sealers: A systematic review

**DOI:** 10.1371/journal.pone.0223575

**Published:** 2019-10-17

**Authors:** Marija Šimundić Munitić, Tina Poklepović Peričić, Ana Utrobičić, Ivona Bago, Livia Puljak

**Affiliations:** 1 Department of Endodontics and Restorative Dentistry, Dental Polyclinic Split; School of Dental Medicine, University of Split, Split, Croatia; 2 Department of Research in Biomedicine and Health, School of Medicine, University of Split, Split, Croatia; 3 Cochrane Croatia, Central Medical Library, School of Medicine, University of Split, Split, Croatia; 4 Department of Endodontics and Restorative Dentistry, School of Dental Medicine, University of Zagreb, Zagreb, Croatia; 5 Department of Evidence Based Medicine and Health Care, Catholic University of Croatia, Zagreb, Zagreb, Croatia; Universite de Technologie de Compiegne, FRANCE

## Abstract

**Background:**

Recently, a new generation of bioceramic root canal sealers has been introduced onto the market. Many in vitro studies have investigated the antimicrobial properties of these sealers, but their comparative efficacy in antimicrobial activity is still unknown.

**Methodology:**

Three electronic databases were searched: MEDLINE and Embase via the OvidSP platform, and Web of Science, up to June 25, 2019. Studies were included irrespective of study design, type of publication and language. Reporting quality was assessed by two authors independently. Meta-analysis was not performed due to studies being highly heterogeneous.

**Results:**

We included 37 studies that analysed the antimicrobial effects of bioceramic sealers. Most of them used a planktonic cell model, with the exception of nine studies which used biofilms. It was not possible to make direct comparison of results from studies and to give a clear conclusion about the comparative antimicrobial activity of these materials because the studies used heterogeneous sources and ages of microorganisms, setting and contact times of sealers, and antimicrobial tests. Furthermore, some materials showed completely different results when tested with different methods.

**Conclusions:**

In conclusion, multiple in vitro studies have shown that bioceramic sealers may have various degrees of antimicrobial activity. However, it is still impossible to make conclusions about their comparative efficacy and to recommend the use of one over another in clinical practice because the studies available were conducted in different ways, which makes meta-analysis futile. A uniform methodological approach, consistent definitions and studies on humans are urgently needed in this field of research so that recommendations for practice can be made.

## 1. Introduction

Microorganisms and their products are the main aetiologic factors responsible for pulpal diseases and periapical lesions [1]. Microorganisms found in root canals are commonly organized in biofilms, in which they are more resistant to antimicrobials than bacteria in the planktonic state [2]. Shen et al. [3] showed that biofilms aged 3 weeks and older are more resistant to chlorhexidine (CHX) than 2-week old and younger biofilms. Similar results were shown by Stojicic, Shen and Haapasalo [4], where 1% sodium hypochlorite (NaOCl) and 2% CHX were effective in killing 1- and 2- week old bacterial biofilms, whereas 3- week- old and older biofilms showed increased resistance to these agents.

The objectives of root canal treatment are elimination of infection from the root canal and prevention of its reinfection by filling and sealing the root canal space [5–7]. Although chemomechanical preparation significantly reduces the number of microorganisms in the root canal, 40–60% of root canals still remain positive for bacterial presence after this treatment [8,9].

Thus, endodontic sealers play an important role in controlling endodontic infection by entombing residual bacteria and preventing leakage of nutrients and reinfection of the root canal [10]. Multiple commercial endodontic sealers, available on the market, are claimed to have antimicrobial properties. Many studies have reported that freshly prepared root canal sealers (resin-, zinc oxide eugenol-, calcium hydroxide-, silicate- and silicon- based sealers) are effective against *Enterococcus faecalis* (*E*. *faecalis*), but their antimicrobial effectiveness after 2 to 7 days of ageing has not been reported [11–16].

Bioceramic materials represent materials based on tricalcium phosphate, mineral trioxide aggregate (MTA) and tricalcium silicate [17].

The first bioceramic material used for root canal obturation was described in 1984 [18]. The forerunners of modern bioceramic materials were calcium phosphate sealers like Sankin apatite root canal sealers (I, II and III) (Sankin Kogyo, Tokyo, Japan) and experimental sealers known as Capseal (I and II) [17].

A new era of bioceramic materials started in the mid- 1990s when bioceramic materials based on MTA were introduced firstly as root repair cements [19]. Those were mainly Portland-derived cements like ProRoot MTA (Dentsply Tulsa, Tulsa, OK, USA), which have been used as root-end filling materials, and root repair and pulp capping materials [20–22]. Because of their dense consistency, these cements are not easy to place in root canals [23], therefore, bioceramic based root canal sealers have recently been developed [24,25]. The first sealer based on MTA was MTA Fillapex (Angelus, Londrina, Brazil), introduced onto the market in 2010. This sealer is composed mainly of a salicylate resin matrix, silica, and MTA (40%).

Another type of bioceramic materials is calcium silicate materials. They contain zirconium oxide, tricalcium silicate, dicalcium silicate, colloidal silica, calcium silicates, monobasic calcium phosphate, and calcium hydroxide [26]. Their pH is above 12, so they have similar antibacterial properties to calcium hydroxide [25,27–29].

As well as for two-components sealers such as Bioroot Root Canal Sealer (BioRoot RCS, Septodont, Saint- Maur- des- Fossés France), premixed bioceramic sealers, e.g. iRoot SP root canal sealer (Innovative BioCeramix Inc., Vancouver, Canada) and Endosequence BC Sealer (Endosequence BC Sealer, Brasseler, Savannah, GA, USA) are available on the market. Bioceramic sealers have also attracted attention because of their alkaline pH, biocompatibility, bioactivity, non-toxicity, dimensional stability, sealing ability and potential to increase root strength after obturation [27,30].

However, the comparative antimicrobial effectiveness of sealers is unknown, and it is not known which models have been used to prove their antimicrobial activity.

Therefore, the aim of this systematic review of the literature was to provide knowledge synthesis of the available body of evidence regarding the antimicrobial properties of endodontic bioceramic sealers for differently aged microorganisms, regardless of study design, and to analyse their methods, results, conclusions and comparative effectiveness, as well as reporting the quality of the literature published on this subject.

## 2. Methods

We conducted a systematic review of literature according to the methods and guidelines from the Centre for Reviews and Dissemination [31] and the PRISMA Statement [32]. The protocol of the systematic review was registered and published in the PROSPERO database (registration number: CRD42018082375).

### 2.1. Clinical question

We defined the clinical question in the following way: i) we included studies that analysed the antimicrobial properties of bioceramic endodontic sealers, conducted on any type of patient or in vivo and in vitro experimental models; ii) eligible interventions were endodontic bioceramic sealers; iii) any type of comparator was eligible; iv) outcomes were size of the inhibition zone, number of microorganisms, percentage of dead cells in dentinal tubules, changes in microbial growth and biovolume of viable cells.

### 2.2. Inclusion criteria

Studies of any design that analysed the antimicrobial properties of endodontic bioceramic sealers, in vivo studies on both humans and animals and in vitro studies conducted on any type of laboratory model were considered for inclusion in this review.

### 2.3. Exclusion criteria

Studies were excluded if they evaluated the antimicrobial properties of other types of root canal sealers (calcium hydroxide, resin, zinc eugenol or silicone- based sealers). Likewise, studies that evaluated bioceramic- based cements such as MTA cements or Biodentin (Septodont, Saint- Maur- des- Fossés, France) were excluded because their use for the purpose of root canal filling is limited. Experimental sealers which are not commercially available on the market were also excluded. Studies that evaluated sealers developed before the MTA era were also excluded.

### 2.4. Search strategy

Three electronic databases were searched: MEDLINE (Ovid MEDLINE(R) Epub Ahead of Print, In Process & Other Non- Indexed Citations, Ovid MEDLINE(R) Daily and Ovid MEDLINE(R)) and Embase (Embase Classic+ Embase) via the OvidSP platform, and the Web of Science Core Collection. Searches were performed without time limit, from the database inception up to June 25, 2019. The search strategy was designed by combining search terms related to bioceramic endodontic sealers with those for antimicrobial activity (S1 Appendix). There were no restrictions regarding the language or status of the publication. Search results were exported into EndNote X5 software (Clarivate Analytics, Boston, MA, USA). Duplicates were removed, at first by using the built-in EndNote feature for de-duplication and then manually. In order to find additional studies that might potentially fulfil the eligibility criteria for inclusion, the references of all included studies were searched, and studies that cited all included studies were also searched at the Web of Science; these were then screened to potentially find more relevant studies that were not found during the initial database search.

### 2.5. Study selection

Two authors (MŠM, TPP) independently screened titles and abstracts obtained via the database search. Full texts of studies that were considered relevant or potentially relevant in the first screening phase were obtained and thoroughly analysed for eligibility by two authors independently (MŠM, TPP). At both stages of the screening process, all discrepancies were resolved via discussion or by involving the third author (IB).

### 2.6. Outcomes

Outcome measures used in this systematic review were as follows: size of the inhibition zone, number of microorganisms (colony- forming units), percentage of dead cells in dentinal tubules, changes in microbial growth and biovolume of viable cells.

### 2.7. Data extraction

After screening the full texts, a data extraction sheet was developed, tested on two studies and refined accordingly. Two authors (MŠM, TPP) independently extracted data. All disagreements were resolved via discussion or by involving the third author (IB). The following data were collected from each study: (i) general information, including the first author’s name, publication year, aim of the study, study design; (ii) general methods: evaluation methods, model used, microorganisms tested, duration of microorganism growth, sealer tested, setting time of sealer before contact with microorganisms, contact time between sealer and microorganisms; (iii) outcomes studied and (iv) experimental results. In the case of incomplete or unclear data, study authors were contacted for clarifications. If authors did not respond after the second email, we did not contact them further.

Studies were then divided into seven groups depending on which material they used. Young and mature biofilms were defined according to the study of Stojicic, Shen and Haapasalo [4] where young biofilms implied only microbial clusters up to 2 weeks old and mature biofilms as bacterial clusters of more than 2 weeks of maturation.

### 2.8. Data synthesis

For all included studies, narrative and tabular synthesis of data was performed. Meta- analysis could not be performed due to the heterogeneity of studies.

### 2.9. Reporting quality of included studies

Reporting quality was assessed by two authors (MŠM, TPP) independently. Model of testing, sample size and suitability description of the sealer were analysed.

## 3. Results

**Fig 1:**
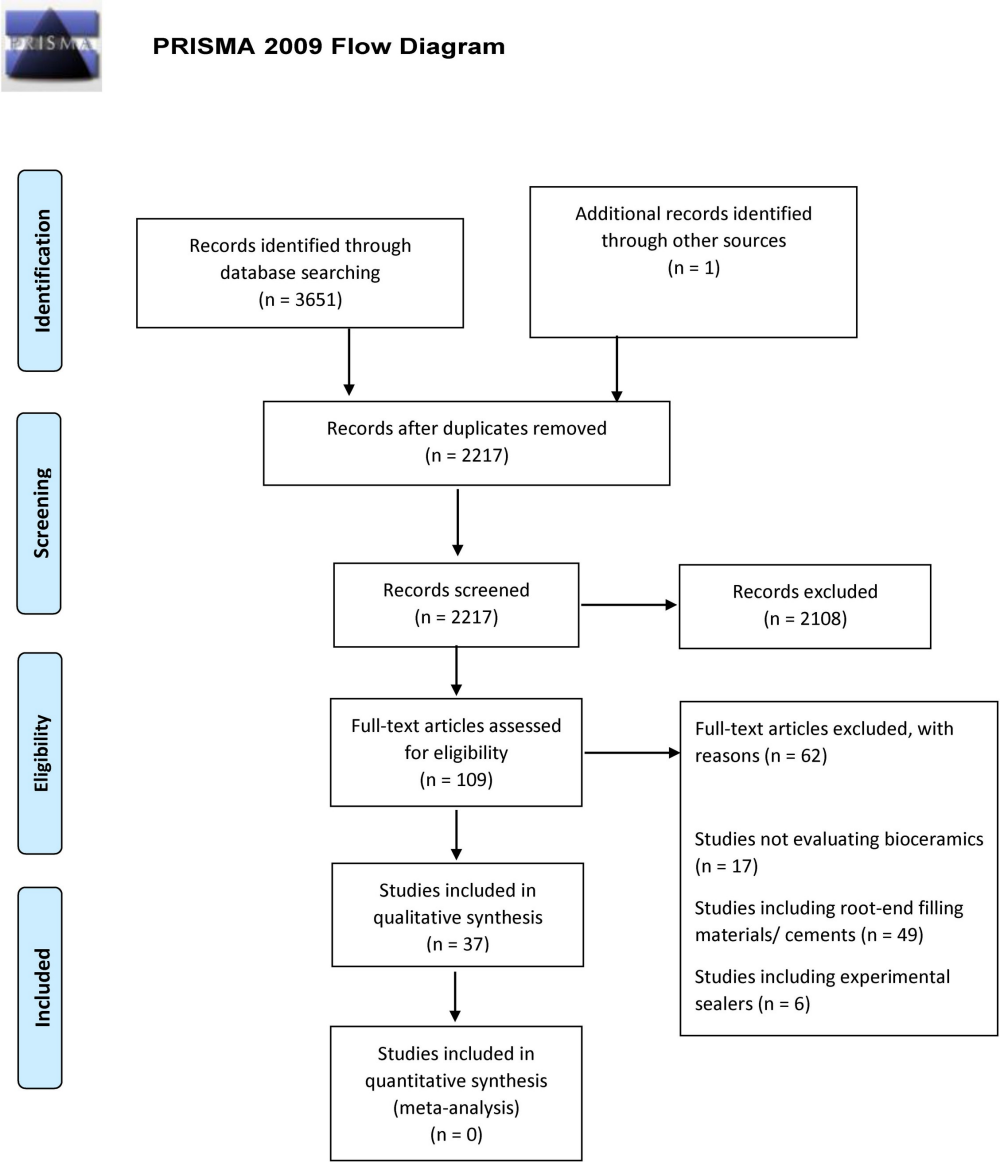
PRISMA 2009 flow diagram.

The study selection flow chart representing the stages of the systematic review process is presented in Fig 1.

The search strategy yielded 3651 results consisting of titles with or without abstracts. After software and manual de-duplication, 2217 were screened. For further inclusion, 109 titles were considered. Then, abstracts and full texts were searched. The final number of included studies, which met the established criteria, was 37 including three studies [33,34,35] of which two [34,35] were published only as a conference abstract, and another [33] which was found by searching other sources. Neither human nor animal in vivo studies were found. The authors had access to all full texts.

### 3.1. Characteristics of included studies

Detailed characteristics of included studies are shown in the supplementary table (https://figshare.com/articles/Antimicrobial_efficacy_of_commercially_available_endodontic_bioceramic_root_canal_sealers/9632075). The following materials were investigated in the included studies: MTA Fillapex, Endosequence Bioceramic Sealer, Totalfill Bioceramic Sealer (Totalfill BC Sealer, Brasseler USA, Savannah, GA, USA), iRoot SP, BioRoot RCS, CPM Sealer (EGEO, Buenos Aires, Argentina) and Smartpaste Bio (Smart Seal DRFP Ltd, Stamford, England). Three studies investigated the combined antimicrobial effect of irrigants and root canal sealers [36–38]. Most studies used a planktonic cell model, with the exception of nine studies which used young biofilms [10,36,38] or mature biofilm [37,39–43] for testing purposes.

One study [44] reported only qualitative results. Thus, data about the antimicrobial efficacy of each group could not be extracted precisely. We contacted the corresponding authors in cases when additional data were required [27,44–53], but most of them did not reply after the second email. One author [47] replied and wrote that disclosure of raw data is against their policy. One message was returned as undelivered [44].

### 3.2. Effectiveness of bioceramic sealers

#### 3.2.1. MTA Fillapex

Most of the studies investigated the antimicrobial efficacy of MTA Fillapex. Nineteen studies used planktonic cells [16,33,34,38,45,46,48,50,52–62] while four studies used either young biofilms [38,49] or mature biofilms [37,40].

The most commonly used antimicrobial test was the agar diffusion test (ADT) [33,34,38,45,48,50,52,53,59–61]. Nine studies used the direct contact test (DCT) [16,36,40,46,50,56–58,62], where colony forming units (CFUs) were counted. Two studies [54,55] used DCT readings of optical density (OD). Only one study [37] used confocal laser scanning microscopy (CLSM) to investigate antimicrobial efficacy. Antibacterial efficacy was studied in 19 studies [16,33,34,36–38,40,46,50,52,53,55–62]. The results of these studies are shown in Table 1.

**Table 1:** Antibacterial efficacy of MTA Fillapex.

Author and the year of study publication	Bacteria used	Evaluation method	Sealer setting time (before contact with bacteria)	Contact time of sealer and microorganisms	Results
Arias-Moliz and Camilleri, 2016 [38]	*E*. *faecalis* (ATCC 29212)	ADT, Intratubular infection test (using CLSM)	ADT 24 h at 37°C in 100% humidity CLSM Freshly mixed sealers	ADT 24 h CLSM 7 days at 37°C in 100% humidity	ADT MTA Fillapex revealed no antibacterial efficacy when exposed to water or PBS. In the EDTA group, MTA Fillapex showed the lowest antibacterial efficacy when compared with BioRoot RCS and AH Plus. CLSM BioRoot RCS exhibited the greatest antimicrobial activity in all irrigation regimes followed by MTA Fillapex.
Colombo et al., 2018 [50]	*E*. *faecalis* (ATCC 29212)	ADT, DCT	ADT Not clearly reported DCT 7 days at 37°C in 100% humidity	ADT 48h DCT 6, 15, and 60 min then 24h at 37°C	ADT MTA Fillapex showed the lowest antibacterial efficacy when compared with EasySeal^(a)^ and AH Plus. MTA Fillapex, BioRoot RCS and Sealapex^(b)^ had comparable antibacterial efficacy. Totalfill showed no antibacterial effectiveness. DCT The best antimicrobial activity against *E*.*faecalis* was shown in Totalfill and EasySeal groups where all bacteria were killed in all contact times. BioRoot RCS and MTA Fillapex showed lower means of the CFUs after 6 min of contact- however, that effect improved after 15 and 60 min and it was higher for BioRoot RCS. ^(a)^ (Komet, Brasseler GmbH & Co., Lemgo, Germany) ^(b)^ (Kerr, Orange, CA, USA)
Dalmia et al., 2018 [53]	*E*. *faecalis* (MTCC2093)	ADT	Freshly mixed sealers	72h at 37°C under aerobic conditions	MTA Fillapex showed the least antibacterial effect when compared with AH Plus, Tubliseal^(c)^ and Sealapex. It also showed a decrease in inhibition zone size over time (the highest after 24h and the lowest after 72h). ^(c)^ (Kerr, Scafati, Italy)
del Carpio-Perochena et al., 2015 [36]	*E*. *faecalis* (ATCC 29212)	DCT and MRT, CLSM	DCT and MRT 7 days CLSM Freshly mixed sealers	DCT and MRT 30 min at 37°C CLSM 7 days at 37°C	DCT and MRT MTA Fillapex showed high antibacterial activity regardless of the addition of Chitosan nanoparticles when it was compared with ThermaSeal^(d)^ (p > 0.05). Insertion of filter membrane reduced the action of sealer (p < 0.05). CLSM After 7 days, MTA Fillapex and ThermaSeal did not show significant difference (p > 0.05). ^(d)^ (Dentsply Tulsa Dental, Tulsa, OK, USA)
Du et al., 2015 [37]	*E*. *faecalis* (VP3- 181)	CLSM	Freshly mixed sealers	7, 30 and 60 days at 37°C in 100% in relative humidity	Significantly more bacteria were dead when NaOCl and sealers (exposure for 30 and 60 days) were used in combination than alone (p < 0.05). After 30- and 60- days of exposure, more dead bacteria were presented than for 7- day exposure (p < 0.05). The combination of NaOCl and MTA Fillapex showed the highest antibacterial effect by reducing 83% of the bacteria (p < 0.05). The difference between sealers with or without NaOCl after 7 days was not statistically significant (p > 0.05).
Faria- Junior et al., 2013 [40]	*E*. *faecalis* (ATCC 29212)	DCT	2 or 7 days	5, 10 and 15 h	Results for setting time of 2 days MTA Fillapex reduced more bacteria when compared with other sealers and the control group at all contact times (p < 0.05). Also, MTA Fillapex showed greater bacterial reduction after 15 h in comparison with the 5 h contact period (p < 0.05). Results for setting time of 7 days No difference was shown between the groups after 5 h (p < 0.05). There was no significant reduction in the number of bacteria in the remaining groups.
Gholamhoseini, Alizadeh and Bolbolian, 2018 [52]	*E*. *faecalis* (ATCC 29212) and *S*. *aureus* (ATCC 25923)	ADT	Freshly mixed sealer	Not clearly reported	Only MTA-Fillapex sealer showed antibacterial effect against *E*. *faecalis*. Against *S*.*aureus*, efficacy of MTA- Fillapex was comparable that of Sure-endo^(e)^. ^(e)^ (Sure-endo, South Korea)
Gürel, 2016 [33]	*E*. *faecalis* (ATCC 29212), *S*. *aureus* (ATCC 29213), *P*. *aeruginosa* (ATCC 27853) and *E*. *coli* (ATCC 25922)	ADT	Freshly mixed sealer	2 h at room temperature then at 37°C for 24, 48 and 72 h	MTA Fillapex showed lower antimicrobial activity in comparison with Smartpaste Bio at all time points and for all bacteria (p < 0.05). Also, AH Plus ^(f)^ showed significantly greater inhibition zones in comparison with MTA Fillapex, except for *E*. *coli* after 72 h. In general, all sealers showed greatest antimicrobial activity after 24 h with decreasing activity after 48 and 72 h for each group. ^(f)^ (Dentsply, DeTrey, Konstanz, Germany)
Hasheminia et al., 2017 [58]	*E*. *faecalis* (PTCC139)	ADT, DCT	ADT Freshly mixed sealer DCT 7 days at 37°C	ADT 2 h at room temperature then 48 h at 37°C DCT 6, 15 and 60 min	ADT MTA Fillapex showed no difference comparing with RoekoSeal sealer (p = 0.99). They revealed the least antibacterial activity. DCT MTA Fillapex showed higher antibacterial activity in comparison with other sealers at all time points (p < 0.05).
Jafari et al., 2016 [55]	*Lactobacillus acidophilus* (*L*. *acidophilus*) (ATCC 4356), *L*. *casei* (ATCC 39392), *S*. *aureus* (ATCC 25923) and *E*. *faecalis* (ATCC 29212)	Contact test (direct and indirect techniques)	Not clearly reported	Not clearly reported	Results are not clearly reported. We analysed results from Tables 1. and 2. Direct method Both sealers (MTA Fillapex and AH 26^(g)^) tested showed a significantly decrease over time for all bacterial species, except for MTA Fillapex on *L*. *acidophilus*, *L*. *casei* and *E*. *faecalis*. MTA Fillapex had a similar effect on *S*. *aureus*, *L*. *acidophilus* and *L*. *casei* and the lowest antibacterial effect on *E*. *faecalis*. In general, the effectiveness of MTA Fillapex was significantly lower than that of AH 26 sealer. Indirect method MTA Fillapex showed the greatest effectiveness on *E*. *faecalis* and the lowest on *L*. *acidophilus*. In general, both sealers had a similar antibacterial effect. ^(g)^ (Dentsply, DeTrey, Konstanz, Germany)
Madani et al., 2014 [46]	*E*. *faecalis* (PTCC 1394), *E*. *coli* (DH5), *S*. *mutans* (PTCC 1683)	DCT	Not clearly reported	1 h for evaporation of microbial suspension then 3,6 and 24 h at 37°C	When compared with AH26, MTA Fillapex was more effective in reducing the number of *E*. *faecalis* and *E*. *coli* after 3, 6 and 24 h and *S*. *mutans* colonies after 24 h.
Morgental et al., 2011 [16]	*E*. *faecalis* (ATCC 29212)	ADT, DCT	ADT Freshly mixed sealer DCT 7 days	ADT 2 h at room temperature then 48 h at 37°C under aerobic conditions DCT 1, 6, 15 and 60 min	ADT MTA Fillapex and Endofill^(h)^ (positive control) had the largest inhibition zone when compared with other sealers (p < 0.05). DCT All sealers were similar to the negative control group at all time periods (p> 0.05). ^(h)^ (Dentsply, Petrópolis, RJ, Brazil)
Nejadshamsi et al., 2017 [60]	*E*. *faecalis* (ATCC 29212)	ADT	Freshly mixed sealers	72h at 37°C	MTAfillapex had the lowest antibacterial effect, which decreased slightly with time.
Nezhadshamsi, Forghan-Parast and Sahranavard, 2014 [34]	*E*. *faecalis* (ATCC 29212)	ADT	Freshly mixed sealers	24, 48 and 72 h	MTA Fillapex showed significantly lower antibacterial efficacy when compared with AH 26 and AH Plus.
Omidi et al., 2018 [61]	*Streptococcus faecalis* ATCC (1394)	ADT	Freshly mixed sealers	24 h on 37°C	MTA Fillapex showed slightly lower antibacterial efficacy than AH 26, but better than AH Plus.
Poggio et al., 2017 [56]	*E*. *faecalis* (ATCC 29212)	ADT, DCT	ADT Not clearly reported DCT 7 days	ADT 2 h at room temperature then 48 h at 37°C DCT 6, 15 and 60 min	ADT MTA Fillapex was comparable with BioRoot^™^ RCS, and Sealapex Root Canal Sealer and they showed the lowest antibacterial activity compared to the others. DCT MTA Fillapex, BioRoot RCS, Pulp Canal Sealer^™(i)^ and N2^(j)^ showed the least means of the CFUs after 6 min of contact. MTA Fillapex showed a significant increase in bactericidal effect (p < 0.05) after 15 and 60 min. ^(i)^ (Kerr, Orange, CA, USA) ^(j)^ (GHIMAS S.p.A, Casalecchio di Reno, BO, Italy)
Prathita, Djauharie and Meidyawati, 2019 [62]	*E*. *faecalis* (ATCC 29212)	DCT	1 and 7 days	1 h at 37°C in 100% humidity	One day after preparation, MTA Fillapex showed the lowest number of CFUs which was better than in the Apexit Plus group. One or seven day old MTA Fillapex had better efficacy than freshly mixed sealer and between these time points there was no significant difference. After 7 days, MTA Fillapex exhibited better efficacy than Apexit Plus.
Shakya et al., 2016 [57]	*E*. *faecalis* (ATCC 29212)	ADT, DCT	Freshly mixed sealer	ADT 7 days DCT 1 and 24h	ADT The MTA Fillapex inhibition zone decreased after 7 days (p = 0.0001). MTA Fillapex had lower efficacy when compared with CRCS (p = 0.0001) and better when compared with AH Plus (p = 0.0001) after 24h. After 7 days, antibacterial efficacy of all sealers decreased, but MTA Fillapex still had higher antibacterial efficacy that of AH Plus. DCT After 1 h, MTA Fillapex provided the greatest decrease in number of bacteria but after 24 h CRCS and MTA Fillapex showed similar results.
Thanish Ahamed and Geetha, 2017 [59]	*E*. *faecalis*	ADT	Not clearly reported	Overnight at 37°C	Results are shown without SDs and p -values. MTA Fillapex revealed similar efficacy when compared with Zinc oxide eugenol and its activity was lower than that of Endomethasone^(k)^. ^(k)^ (Septodont Saint-Maur-des-Fossés, Cedex, France)

The antifungal efficacy of MTA Fillapex was studied in five studies [33,45,46,48,54] and the results are shown in Table 2.

**Table 2:** Antifungal efficacy of MTA Fillapex.

Author and year of study publication	Fungi used	Evaluation method	Sealer setting time (before contact with fungi)	Contact time of sealers and microorganisms	Results
Gürel, 2016 [33]	*C*. *albicans* (ATCC 10231)	ADT	Freshly mixed sealer	2 h at room temperature then 24, 48 and 72 h at 37°C.	Smartpaste Bio showed lower inhibition zones than MTA Fillapex at all time points (p < 0.05). Also, AH Plus showed significantly greater inhibition zones in comparison with MTA Fillapex after 72 h. Each root canal sealer had strongest antimicrobial activity at 24 h and the lowest antimicrobial activity at 72 h.
Jafari et al., 2017 [54]	*C*. *albicans* (ATCC 10231), *C*. *glabrata* (ATCC 90030) and *C*. *krusei* (DSM 70079)	Contact test- (direct and indirect methods)	Not clearly reported	Not clearly reported	Direct method MTA Fillapex sealer showed the highest effect on *C*. *albicans* and the lowest on *C*. *krusei*. AH 26 had significantly better efficacy on *C*. *krusei* and *C*. *glabrata* than MTA Fillapex. Indirect method MTA Fillapex and AH 26 showed similar effectiveness, althought results were not statistically significant.
Madani et al., 2014 [46]	*C*. *albicans* (PTCC 5027)	DCT	Not clearly reported	1 h for evaporation of microbial suspension then 3, 6 and 24h at 37°C	When compared with AH26, MTA Fillapex was more effective in reducing the number of *C*. *albicans* colonies after 6 and 24 h.
Oczan et al., 2013 [45]	*C*. *albicans* (ATCC 10231)	DCT	20 min, 1 and 7 days	1 h	MTA Fillapex and iRoot SP showed similar results after 20 min of setting (p < 0.05). They were better than GuttaFlow (p < 0.05) while AH Plus showed the greatest reduction inhibiting fungal growth completely after 20 min of setting time. There was no difference between sealers after 1 or 7 -days of setting (p > 0.05).
Weckwerth et al., 2015 [48]	*C*. *albicans* (ATCC 10231)	ADT	Freshly mixed sealer	2 h at room temperature then 24 h at 37°C	MTA Fillapex with addition of ketoconazole and fluconazole presented greater inhibition zones compared to the pure sealer (p < 0.05).

It was not possible to make a uniform conclusion for this group of studies about the comparative efficacy of MTA Fillapex in terms of its antibacterial and antifungal effects because the studies were highly heterogeneous–using different comparator materials, different research methods and different microbial species.

#### 3.2.2. Endosequence BC Sealer, iRoot SP and Totalfill BC Sealer

After the introduction in 2009 of Endosequence BC Sealer, also known as iRoot SP [63], to the North American market, Totalfill BC Sealer, a material with the same composition, was introduced in Switzerland for the European market [64]. Since these three sealers have the same composition, we considered their antimicrobial efficacy as being comparable.

The antimicrobial efficacy of Endosequence BC Sealer was studied in nine studies [35,37,39,42,44,51,65–67]. Six of them investigated efficacy on planktonic cells [35,44,51,65–67] and three on mature biofilms [37,39,42].

In three studies, [65–67] ADT was used to investigate the antimicrobial efficacy of Endosequence BC, and three studies used CLSM [37,39,42]. Also, three studies used DCT [35,51,65] and one used scanning electron microscopy (SEM) evaluation [44].

Two studies investigated the antibacterial efficacy of iRoot SP. One of them [27] studied only efficacy against *E*. *faecalis*, and another against *E*. *faecalis* and *Staphylococcus aureus* (*S*. *aureus*) [47]. Antifungal activity was studied in two studies [45,47]. All studies were conducted on planktonic cells [27,45,47].

DCT was used in the studies of Ozcan et al. [45] and Nirupama et al. [47] and a modified direct contact test (MDCT) was used in the study of Zhang et al. [27].

Five studies investigated the antimicrobial efficacy of Totalfill BC sealer [10,41,43,50,56]. Two studies [50,56] used planktonic cells in ADT and DCT. Kapralos et al [10] used planktonic cells in MDCT and young biofilms in DCT and the membrane restricted test (MRT). Zordan-Bronzel et al [43] used planktonic cells in DCT and old biofilm in MDCT, whereas Alsubait et. al. [41] used old biofilms in CLSM.

Willershausen et al. [44] used SEM to explore bacterial growth, but there was no control group and the results were not clearly reported.

The results of all studies that analysed the antibacterial activity of Endosequence BC Sealer, iRoot SP and Totalfill BC Sealer are shown in Table 3.

**Table 3:** Antibacterial efficacy of Endosequence BC Sealer, iRoot SP and Totalfill BC Sealer.

Author and year of study publication	Bacteria used	Evaluation method	Sealer setting time (before contact with bacteria)	Contact time of sealer and microorganisms	Results
Alsubait et al., 2019 [41]	*E*. *faecalis* (ATCC 47077)	CLSM	Freshly mixed sealer	1, 7 and 30 days at 37°C in 100% humidity	Antibacterial efficacy of AH Plus, Totalfill and BioRoot RCS was comparable after 1 day. Totalfill showed the highest number of dead bacteria after 7 days when compared to days 1 and 30. After 7 days, Totalfill killed significantly more bacteria than in the control group (p = 0.013) and BioRoot RCS (p = 0.000). However, after 30 days of exposure, all sealers killed more bacteria than the control group (p < 0.05) but BioRoot RCS killed a significantly higher (p = 0.04) percentage (61.75%) than Totalfill and AH Plus (p = 0.000).
Brezic et al., 2017 [35]	*Streptococcus mitis* and *Streptococcus oralis*	ADT, DCT	ADT Freshly mixed sealers DCT Not clearly reported	ADT 24h DCT 1, 6, 20 and 24h	ADT Endosequence had the best effect on *S*. *oralis* and N2 against *S*. *mitis*. DCT MTA had the best effect against *S*. *oralis* and AH Plus against *S*. *mitis* after 24 h.
Bukhari and Karabucak, 2019 [42]	*E*. *faecalis* (OG1RF)	CLSM	Freshly mixed sealer	24 h and 2 weeks	Endosequence BC Sealer was superior in killing *E*. *faecalis* compared with AH Plus at both time periods, 2 weeks and 24 h, with a statistically significant difference (p < 0.0005). There was no significant difference between 24 h and 2- weeks group within the Endosequence group (p > 0.05).
Candeiro et al., 2015 [65]	*E*. *faecalis* (ATCC 29212)	ADT, DCT	Freshly mixed sealer	ADT 2 h on room temperature then 48 h at 37°C DCT 7 days at 37°C	ADT The inhibition zone of the AH Plus sealer was greater than in the EndoSequence BC sealer group (p < 0.05). DCT Endosequence BC sealer showed better effectiveness only after 24 h (p < 0.05).
Colombo et al., 2018 [50]	*E*. *faecalis* (ATCC 29212)	ADT, DCT	ADT Not clearly reported DCT 7 days at 37°C in 100% humidity	ADT 48 h DCT 6, 15, and 60 min then at 37°C for 24 h	ADT Totalfill killed no bacteria. DCT Totalfill killed all bacteria.
Du et al., 2015 [37]	*E*. *faecalis* (VP3-181)	CLSM	Freshly mixed sealers	7, 30 and 60 days	Sealers in combination with NaOCl showed better effectiveness in reducing the number of living bacteria. There was no difference between AH Plus and Endosequence BC Sealer (p > 0.05).
Kapralos et al., 2018 [10]	*E*. *faecalis* (ATCC 19434), *S*. *mutans* (ATCC 700610), *Streptococcus epidermidis* (ATCC 35984), and *S*. *aureus* *Newman*	MDCT, DCT and MRT	MDCT Freshly mixed (setting times for the freshly mixed samples for AH Plus were 20 min, for RoekoSeal 50 min and for Guttaflow 2 30 minutes) or after 24h or after 7 days DCT and MRT Freshly mixed sealers	MDCT 1 h at 37°C DCT and MRT 24 h.	MDCT Either freshly mixed or after 24 h and 7 days, TotalFill BC sealer exhibited antibacterial activity for all conditions investigated. *S*. *aureus* was more resistant in water conditions to TotalFill BC sealer compared with the other bacterial species (p < 0.05) DCT and MRT Although, TotalFill BC sealer reduced the number of viable bacteria for all monospecies biofilms (p < 0.05), AH Plus had higher activity against all biofilms when MRT was used. When DCT was used, AH Plus shower higher activity against *S*. *aureus* and *E*. *faecalis* biofilms compared with TotalFill BC sealer.
Nirupama et al., 2014 [47]	*E*. *faecalis* (ATCC 29212) and *S*. *aureus* (ATCC 25923)	DCT	Freshly mixed sealers (20 min)	1 h then bacterial growth was measured every 30 min for 18 h	IRoot SP showed inhibition of *E*. *faecalis* growth only in the first 8 h and *S*. *aureus* growth only in the first 7 h.
Poggio et al., 2017 [56]	*E*. *faecalis* (ATCC 29212)	ADT, DCT	ADT Not clearly reported DCT 7 days	ADT 2 h at room temperature then 48 h at 37°C DCT 6, 15 and 60 min	ADT TotalFill BC Sealer did not cause an inhibition zone. DCT TotalFill BC Sealer killed all bacteria in all time periods.
Shin, Lee and Lee, 2018 [51]	*E*. *faecalis* (ATCC 29221) , *P*. *endodontalis* (ATCC 35406) and *P*. *gingivalis* (ATCC 33277)	DCT	Freshly mixed sealer and after 24 h at 37°C with agitation for 4 h	24 h at 37°C	In the *E*. *faecalis* group, freshly mixed Endosequence showed the lowest antibacterial activity, and no antibacterial activity when material was set. In *P*. *endodontalis* and *P*. *gingivalis* groups, Endosequence exhibited the lowest antibacterial activity regardless of whether the material was set.
Singh, Gupta et al., 2016 [66]	*E*. *faecalis* (ATCC 29212)	ADT	Freshly mixed sealer	2 h at room temperature then 24 h at 37°C	Endosequence BC Sealer showed the largest inhibition zone, but the observed advantage in relation to ProRoot WMTA and MM-MTA^(i)^ was not statistically significant (p > 0.05). ^(i)^ (Micro Mega, Besançon, France)
Singh, Elshamy et al., 2016 [67]	*Lactobacillus*, *S*. *aureus*, *E*. *coli*, *P*. *aeruginosa*	ADT	Freshly mixed sealer	2 h at room temperature then 24 h at 37°C	Endosequence BC sealer showed the greatest inhibition zones against all the microorganisms but the difference was not statistically significant (p > 0.005).
Wang, Shen and Haapasalo, 2014 [39]	*E*. *faecalis* VP3-181	CLSM	Freshly mixed sealers	1, 7 and 30 days	All sealers killed more bacteria than the control group at all time periods (p < 0.05). The antibacterial activity of Endosequence BC sealer increased over time (p< 0.05). There was no difference between Endosequence BC sealer and AH Plus (p > 0.05).
Zhang et al., 2009 [27]	*E*. *faecalis* (VP3-181), isolated from a case of persistent apical periodontitis	MDCT	20 min, 1, 3 and 7 days	2, 5, 20 and 60 min at 37°C at 100% humidity	Freshly mixed iRoot SP killed all bacteria within 2 min of contact, after 1 day of setting iRoot reduced the number of bacteria significantly (p < 0.05) during the first 2 min while all bacteria were killed within 20 min. IRoot had stable effectiveness for up to 3 days, but after 7 days it lost its efficacy.
Zordan- Bronzel et al., 2019 [43]	*E*. *faecalis* (ATCC 29211)	DCT and MDCT	DCT 24 h MDCT Not clearly reported	DCT 1 h and 30 min MDCT 15 h	DCT Totalfill reduced the number of *E*. *faecalis* significantly when compared with the control group (p < 0.05). MDCT Totalfill showed significantly higher effectiveness against *E*. *faecalis* when compared with AH Plus and the control group (p < 0.05).

It was not possible to make a uniform conclusion for this group of studies about the comparative efficacy of Endosequence BC Sealer, iRoot SP and Totalfill BC Sealer in terms of their antibacterial activity because the studies were highly heterogeneous–using different comparators, different research methods and different bacterial species.

Fungi, namely *Candida albicans* (*C*. *albicans*) were included in four studies [43,45,47,67]. In the study of Singh et al. [67] Endosequence BC Sealer showed the largest inhibition zone when compared with MM Seal (Micro Mega, France) and Zical (Prevest DenPro, Jammu, India).

Ozcan et al. [45] showed that freshly mixed iRoot SP produced a significant (p < 0.05) reduction in fungal growth which was not significantly different (p > 0.05) from that in the MTA Fillapex group. IRoot SP showed significantly better results when compared with freshly mixed GuttaFlow (Coltène-Whaledent, Langenau, Germany) (p < 0.05). Only freshly mixed AH Plus showed significantly higher antifungal efficacy than other sealers (p < 0.001). One and seven day old samples exhibited slight or no antifungal efficacy without significant differences between sealers and the positive control (p > 0.05). In the study of Nirupama et al. [47], iRoot SP was comparable with TubliSeal EWT and AH Plus and they had significant antifungal activity when compared to the positive control (p < 0.05).

In the study of Zordan- Bronzel et al. [43], Totalfill completely eliminated *C*. *albicans*.

#### 3.2.3. BioRoot RCS

Four studies [38,41,50,56] investigated the antibacterial efficacy of BioRoot RCS. All studies used *E*. *faecalis*. Two of them [50,56] studied efficacy on planktonic cells, and one [38] was conducted on planktonic cells and young biofilms while one studied efficacy on old biofilms [41]. As mentioned, Poggio et al. [56] and Colombo et al. [50] used DCT and ADT. In the study of Poggio et al. [56], BioRoot RCS exhibited a similar inhibition zone to those of MTA Fillapex and Sealapex Root Canal Sealer in ADT, which was the smallest when compared with Pulp Canal Sealer EWT, AH Plus, N2 and EasySeal sealers. Only Totalfill exhibited no inhibition zone at all. In DCT, the efficacy of BioRoot RCS was comparable with that of MTA Fillapex, Pulp Canal Sealer EWT and N2 and they showed the smallest mean numbers of colonies formed after 6 min of contact. BioRoot RCS also exhibited a significant increase in bactericidal effect (p < 0.05) after 15 and 60 min. In this test, only Totalfill and EasySeal killed all bacteria.

In the study of Colombo et al [50], BioRoot RCS showed the lowest antibacterial activity which was comparable with that of MTA Fillapex and Sealapex in ADT. Only EasySeal showed significantly higher efficacy compared to other sealers (p < 0.01). In DCT, BioRoot RCS showed the lowest activity after 6 min of contact, similar only to MTA Fillapex. Also, after 15 and 60 min, BioRoot RCS showed a significant increase in bactericidal effect (p < 0.05).

In the study of Arias-Moliz and Camilleri [38], ADT and intratubular infection tests (using CLSM) were used. Irrigation with ethylenediaminetetraacetic acid (EDTA) in combination with sealing with BioRoot RCS or AH Plus showed a significantly larger zone of inhibition against planktonic cells than MTA Fillapex in ADT. No inhibition zone was obtained when BioRoot RCS was exposed to phosphate- buffered saline (PBS) or water. In the intratubular infection test, BioRoot showed the highest antibacterial efficacy in all irrigation protocols. Irrigation with EDTA exhibited the highest number of dead cells, followed by water, without significant differences.

In the study of Alsubait et al [41], BioRoot RCS did not significantly differ from AH Plus and Totalfill after 1 day. After 7 days, BioRoot RCS showed the lowest antibacterial activity when compared with Totalfill and AH Plus. However, after 30 days, BioRoot RCS killed the highest percentage of bacteria which was significantly higher than in AH Plus (p = 0.000) and Totalfill groups (p = 0.04).

#### 3.2.4. CPM sealer

The antimicrobial efficacy of CPM Sealer was studied in three studies [16,68,69]. Only Tanomaru et al. [69] investigated the antifungal efficacy of CPM on *C*. *albicans*. Other microorganisms in the same study were: *Micrococcus luteus* (*M*. *luteus*), *S*. *aureus*, *Pseudomonas aeruginosa* (*P*. *aeruginosa*) and *E*. *faecalis*. Mohammadi et al. [68] investigated antibacterial efficacy on *Streptococcus mutans* (*S*. *mutans*) and *S*. *aureus*, while Morgental et al. [16] used *E*. *faecalis*.

All studies used ADT while Morgental et al. [16] used DCT.

In the study of Morgental et al. [16], CPM Sealer in ADT was not able to inhibit *E*. *faecalis* as well as White MTA (Angelus, Londrina, Brazil). A greater inhibition zone was obtained in MTA Fillapex and Endofill (Dentsply, Petrópolis, Brazil) groups. As for DCT, all sealers were similar to the negative control in all experimental periods (p > 0.05).

Tanomaru et al [69] reported mean inhibition zones for six different materials, one of which was CPM. However, statistical analysis was not performed due to different degrees of diffusion in agar among the different materials. Thus, it was not possible to compare the sealers investigated.

The results of the study of Mohammadi et al. [68] are not clear because the data for two sealers that were not previously reported in the methodology are described in the results section.

#### 3.2.5. Smartpaste Bio

Smartpaste Bio was studied in just one study [33]. The microorganisms tested were: *E*. *faecalis* (ATCC 29212), *S*. *aureus* (ATCC 29213), *P*. *aeruginosa* (ATCC 27853), *Escherichia coli* (*E*. *coli*) (ATCC 25922) and the fungus *C*. *albicans* (ATCC 10231). The test used in the study was ADT. Smartpaste Bio showed significant inhibition of bacterial growth (p < 0.05) at all time points, except on *P*. *aeruginosa* where AH plus showed better efficacy. MTA Fillapex showed significantly lower antimicrobial efficacy (p < 0.05) than Smartpaste Bio. All sealers tested showed decreased antimicrobial activity after a prolonged time period.

### 3.3. Reporting quality of literature

Seven studies [48,54,55,59,66,68,69] provided unclear descriptions of the model and the sample size. Two studies [44,47] did not provide a clear report of the sample size, and in five studies [43,50,51,53,67] the experimental model was not described in sufficient detail. Two of the included studies [34,35] were available as an abstract only- therefore, assessment of their reporting quality was not possible. The remaining studies provided clear descriptions of models and sample size [10,16,27,33,36–42,45,46,52,56–58,60–62,65]. All studies adequately reported the sealers used in the study.

## 4. Discussion

We found 37 studies about the antimicrobial efficacy of bioceramic root canal sealers. However, despite this large number of studies, it was not possible to make conclusions about the comparative efficacy of bioceramic sealers because these studies were highly heterogeneous. Since these studies used different sources and ages of microorganisms, different setting and contact times of sealers and different antimicrobial tests, they could not be directly compared, even when they studied the same bioceramic sealers. We were unable to find two studies which used exactly the same experimental conditions, and therefore we were only able to conduct a narrative analysis. Even though this kind of evidence precludes making any conclusions for practice, that could help practitioners in choosing the best bioceramic sealer, our study has unearthed a number of issues that warrant further attention for researchers in this field.

Firstly, there are different classifications of bioceramic root canal sealers. Although many studies investigated these materials and described their compositions, we found only two reviews [17,70] where their classification was suggested, and in these two the classifications were different. Al-Haddad and Che Ab Aziz [17] divided bioceramic materials into three subgroups: calcium silicate-, MTA- and calcium- phosphate based materials, while Jafari and Jafari [70] described only two subgroups: calcium- silicate based (MTA- and non- MTA- based) and calcium- phosphate based materials. We recommend clear classification in order better understand bioceramic materials.

Moreover, although several systematic reviews [11,70–72] discussed the antimicrobial efficacy of bioceramic sealers, none of them provided a broader view of antimicrobial activity. Alshwaimi et al. [11] included only studies on *E*. *faecalis* where DCT was used while Almeida et al. [71] included only studies which compared the antimicrobial activity of bioceramic and conventional materials. Also, Jafari and Jafari [70] and Donnermeyer et al. [72] provided little information about antimicrobial activity.

Secondly, most of the included studies investigated the antimicrobial efficacy of a single microorganism—*E*. *faecalis*, because of its ability to penetrate deep into dentin tubules, form biofilms, survive nutrition deprivation and resist commonly used disinfection agents [73–78]. Also, results from earlier studies suggest that fungi [79,80] could be associated with persistent apical periodontitis, but only a few studies investigated the influence of bioceramic sealers on fungi. Therefore, a recommendation for further studies is to investigate the efficacy of root canal sealers on fungi and on other bacteria lineage which may also be responsible for the failure of root canal treatment [10,43,44,46,47,55,67–69].

Furthermore, despite recent recommendations from 2012. by De Deus [81], published as an Editorial in *International Endodontic Journal*, to use only mature biofilms in such studies, only six [37,39–43] of the included studies investigated the antimicrobial effectiveness of bioceramic sealers on mature biofilms. In this review, we defined young and mature biofilms according to the study of Stojicic, Shen and Haapasalo [4] but there remains need for general consensus on a suitable model of endodontic biofilm age still remains for future studies.

De Deus [81] also recommended that the conditions used should be similar to those in the filled root canal. Hence, older tests like ADT and DCT should be replaced with newer methodology.

It has already been shown that ADT has limitations- such as dependency on the solubility and diffusion characteristics of the test material and media, and it has been proposed that it is only used to test water- soluble materials [58]. However, it is still widely used, as shown in many studies we included [16,34,35,38,48,50,52,53,56–58,60,61,65,66,68,69]. Another commonly used test was DCT. Its limitations are an inability to use freshly mixed sealers because they may adhere to substrate [40], and it does not allow evaluation of microorganisms in biofilms [47,82]. Recently, new technology using CLSM has been introduced [37–39,41,42]. Used with bacterial viability staining, this model might be suitable for measuring the antimicrobial activity of root canal sealers in infected dentin against microorganisms associated in biofilm [39].

It is also worth emphasizing that the included studies used different setting times of sealers and contact times between sealers and microorganisms. It would be worthwhile defining time points within different stages of material setting and important points during contact time. However, it is also disputable whether certain tests could be performed for a prolonged period when it is known that microorganisms could die spontaneously due to environmental conditions [37].

In conclusion, multiple in vitro studies have shown that bioceramic sealers may have various degrees of antimicrobial activity. However, it is still impossible to make conclusions about their comparative efficacy and to recommend the use of one over another in clinical practice because the studies available were conducted in different way, which makes meta-analysis futile. A uniform methodological approach, consistent definitions and studies on humans are urgently needed in this field of research so that recommendations for practice can be made.

## Supporting information

S1 Appendix Search strategies. (XLSX)

## Abbreviations

ADT: agar diffusion test
*C. albicans*: *Candida albicans*
CFU: colony forming units
CHX: chlorhexidine
CLSM: confocal laser scanning microscopy
DCT: direct contact test
*E. coli*: *Escherichia coli*
EDTA: ethylenediaminetetraacetic acid
*E. faecalis*: *Enterococcus faecalis*
MDCT: modified direct contact test
*M. luteus*: *Micrococcus luteus*
MRT: membrane restricted test
MTA: mineral trioxide aggregate
NaOCL: sodium hypochlorite
OD: optical density
*P. aeruginosa*: *Pseudomonas aeruginosa*
PBS: phosphate buffered saline
*S. aureus*: *Staphylococcus aureus*
SEM: scanning electron microscopy
*S. mutans*: *Streptococcus mutans*
